# Differential roles of brain oscillations in numerical processing: evidence from resting-state EEG and mental number line

**DOI:** 10.3389/fnhum.2024.1357900

**Published:** 2024-06-21

**Authors:** Saied Sabaghypour, Farhad Farkhondeh Tale Navi, Nooshin Basiri, Fereshteh Shakibaei, Negin Zirak

**Affiliations:** ^1^Department of Cognitive Neuroscience, Faculty of Education and Psychology, University of Tabriz, Tabriz, Iran; ^2^Safahan Institute of Higher Education, Isfahan, Iran; ^3^Behavioral Science Research Center, Isfahan University of Medical Sciences, Isfahan, Iran; ^4^Faculty of Education and Psychology, University of Tabriz, Tabriz, Iran

**Keywords:** resting-state EEG, mental number line, emotional traits, frontal asymmetry, direction bias, absolute error

## Abstract

Recent works point to the importance of emotions in special-numerical associations. There remains a notable gap in understanding the electrophysiological underpinnings of such associations. Exploring resting-state (rs) EEG, particularly in frontal regions, could elucidate emotional aspects, while other EEG measures might offer insights into the cognitive dimensions correlating with behavioral performance. The present work investigated the relationship between rs-EEG measures (emotional and cognitive traits) and performance in the mental number line (MNL). EEG activity in theta (3–7 Hz), alpha (8–12 Hz, further subdivided into low-alpha and high-alpha), sensorimotor rhythm (SMR, 13–15 Hz), beta (16–25 Hz), and high-beta/gamma (28–40 Hz) bands was assessed. 76 university students participated in the study, undergoing EEG recordings at rest before engaging in a computerized number-to-position (CNP) task. Analysis revealed significant associations between frontal asymmetry, specific EEG frequencies, and MNL performance metrics (i.e., mean direction bias, mean absolute error, and mean reaction time). Notably, theta and beta asymmetries correlated with direction bias, while alpha peak frequency (APF) and beta activity related to absolute errors in numerical estimation. Moreover, the study identified significant correlations between relative amplitude indices (i.e., theta/beta ratio, theta/SMR ratio) and both absolute errors and reaction times (RTs). Our findings offer novel insights into the emotional and cognitive aspects of EEG patterns and their links to MNL performance.

## 1 Introduction

Numerical cognition is important in everyday life. It involves the capability to distinguish between numerical magnitudes and to contemplate this conscious awareness along with the skill of counting, which are important abilities for representing numbers mentally (Nieder, 2005). However, although number magnitude and counting stand out as prominent numerical properties (Shepard et al., 1975), the human understanding of numbers is not limited to such attributes. Various other characteristics of how numbers are mentally represented have been noted to influence one’s abilities in numerical tasks. Many studies that explored numerical representations assume the existence of a mental number line (MNL), wherein numbers and quantities are organized spatially. A well-established tradition suggests that individuals translate numerical representations into corresponding spatial positions (Dehaene et al., 1993; Fias, 1996; Gevers et al., 2006; Nourouzi Mehlabani et al., 2020). Given the importance of performance in MNL for mathematical achievements (Simms et al., 2016; Nazari et al., 2021), investigating the MNL associations with cognitive and emotional factors is important for understanding the neural underpinnings of numerical cognition. Recent research has highlighted the significance of distinct brain oscillations in understanding the neural dynamics of numerical processing using electroencephalography (EEG). These oscillations are also considered to have functional significance in cognitive and emotional processes (Ray and Cole, 1985; Bell and Diaz, 2012). The resting-state (rs) EEG involve characterizing specific EEG signals and reflect the intrinsic properties of neural systems (Yedukondalu and Sharma, 2023b). Understanding the links between rs-EEG and performance in MNL holds promise in elucidating the neural correlates underlying spatial-numerical associations. These insights can pave the way for establishing an empirical association between rs-EEG with respect to general emotional and cognitive traits, on the one hand, and aspects of numerical cognition, on the other. The aim of the current work was to offer insights into the correlation between rs- EEG cognitive and emotional trait measures in spatial-numerical associations.

Various approaches are employed to investigate the spatial-numerical associations, such as magnitude classification (Wood et al., 2008), the spatial-numerical associations of response codes (SNARC) effect (Dehaene et al., 1993), and spatial biases in mathematics (Pinhas and Fischer, 2008). MNL suggests that individuals mentally represent numbers on a line, with smaller numbers located to the left and larger numbers located to the right (Dehaene et al., 1993; Izard and Dehaene, 2008). The number-to-position (NP) task stands as a commonly utilized method for exploring how numbers are represented on the MNL (Siegler and Opfer, 2003; Berteletti et al., 2010; Nourouzi Mehlabani et al., 2020; Nazari et al., 2021). The NP task involves presenting a number line with marked endpoints (such as 0–100) alongside a designated target number. During each trial, participants are required to identify and mark the position of the target number on the number line. Research findings suggest that healthy adults demonstrate a tendency toward a leftward bias in spatial-numerical associations (Lourenco and Longo, 2009), which contrasts with observations influenced by various disorders (Cavézian et al., 2007; Förster et al., 2011; Barker et al., 2019).

Previous works suggested that individuals with right parietal lesions, combined with persistent left neglect, demonstrate a rightward bias in tasks involving the MNL. Specifically, when identifying the midpoint between two numbers, these patients tend to overestimate the midpoint within a given number pair (Zorzi et al., 2002; Rossetti et al., 2004). Also, a study involving a patient with right neglect has corroborated these findings, suggesting the potential parallelism between the spatial attention system utilized in MNL tasks (Pia et al., 2009). In contrast to individuals with unilateral right brain lesions, healthy adults exhibit a leftward bias in MNL (Loftus et al., 2009; Lourenco and Longo, 2009). This consistent leftward inclination is termed pseudoneglect (Bowers and Heilman, 1980a; Jewell and McCourt, 2000). It is suggested that hemispheric asymmetries in spatial attention occur in such direction biases. Moreover, following transcranial magnetic stimulation (TMS) of the right posterior parietal cortex, healthy adults showed a rightward bias (Fierro et al., 2001). These findings point to the special role of the parietal cortex in modulating attention that might lead to directional biases in MNL. However, our recent findings pointed to the importance of the emotional valence of the participants in MNL direction bias (Sabaghypour et al., 2023). Specifically, we uncovered evidence showing a distinct impact of emotional valence (using IAPS-based film clips inducing positive and negative emotions), on participants’ numerical estimations and spatial representations along the MNL. The investigation revealed that individuals experiencing positive emotional valence exhibited a notable rightward bias in their numerical estimations, whereas those in the negative valence group demonstrated a leftward bias. These insights expand upon previous studies suggesting a connection between emotions and numerical cognition (De Smedt et al., 2009; Holmes and Lourenco, 2011; Schukajlow et al., 2017; Fischer et al., 2021), highlighting the possible links between affective traits and spatial-numerical associations.

As per the valence hypothesis, the right hemisphere mediates negative emotions like sadness, while the left hemisphere deals with positive emotions like happiness (Borod et al., 1998; Martin and Altarriba, 2017; Roesmann et al., 2019; Sabourimoghadam et al., 2022). Consequently, people tend to associate positive emotions with the right side and negative emotions with the left side of the space (Casasanto, 2009; Freddi et al., 2016). Hence, based on the valence hypothesis, negative emotions might bias attention toward the left hemispace and positive emotions might bias attention more to the right (Foster et al., 2008). In addition, animal studies also demonstrate preferences in two crucial survival activities (Rogers, 2017). Feeding tendencies lean toward right-sided behavior, influenced by the left hemisphere (Andrew et al., 2000; Karenina et al., 2016). Conversely, avoiding predators is associated with behavior regulated by the right hemisphere (Robins and Rogers, 2004; Koboroff et al., 2008). Furthermore, a distinct association linking numbers and space in a left-to-right direction has been evidenced in non-human animals (Rugani et al., 2007, 2011; Adachi, 2014). Hence, it has been suggested that changes in numerosity toward larger or smaller magnitudes might be associated with increased activity in the left (positive valence) or right (negative valence) hemisphere (Vallortigara, 2018). Investigating the frontal asymmetry in different frequency bands [i.e., theta (3–7 Hz), alpha (8–12 Hz), and beta (13–25 Hz)] might advance this proposal by providing links to neural activities that may correlate with direction bias in MNL in humans. ‘Asymmetry’ is defined by measurements taken from both sides, with the difference between these measurements serving as an index of the difference between two hemispheres.

Moreover, frontal rs-EEG might be a good predicator of emotional processes (Jackson et al., 2000, 2003; Campagna et al., 2021). Neuroimaging research also suggests that individual differences in emotional traits may be reliably indexed by prefrontal activity at rest (Paradiso et al., 1995; Jackson et al., 2000; Dalton et al., 2002; Zhang et al., 2020). According to the research on frontal EEG asymmetry, increased left prefrontal activity—indexed by alpha and increased beta activity (e.g., at the F3 electrode)—is correlated with positive emotions. As well, increased left frontal theta activity is linked to negative emotional processing (Dharmadhikari et al., 2018; Koller-Schlaud et al., 2020). Conversely, elevated right prefrontal activity (e.g., at the F4 electrode) is associated with negative emotions (Jackson et al., 2003; Reznik and Allen, 2018). Therefore, exploring how this functional hemispheric imbalance correlates with direction bias in spatial-numerical associations could offer an intriguing path for understanding how the brain’s asymmetrical activity, particularly in the frontal regions, is linked to performance in MNL.

In addition to its role in emotional processes, EEG findings also shed light on the correlation between specific frequency bands and cognitive processing, emphasizing the importance of distinct EEG measures (Yedukondalu and Sharma, 2023a; Jain et al., 2024; Yedukondalu et al., 2024). For instance, the theta/beta ratio (TBR) at central areas (like the Cz electrode) (Clarke et al., 2019), and the individual frontal alpha peak frequency (APF) as trait-like parameters of EEG (Sambrook and Goslin, 2015), could serve as indicative measures of cognitive processing capacity and performance (Finley et al., 2022). Alpha frequency is also divided into 8–10 Hz (low alpha) and 10–12 Hz (high alpha), allowing for comparison between these sub-bands (Pfurtscheller, 1989; Zoefel et al., 2011). This classification relies on the assumption that alpha rhythms stem from at least two separate neural groups, one oscillating below 10 Hz and the other above 10 Hz. Regarding the amplitude of sub-alpha bands, results revealed that high alpha interacts with visual cognitive tasks and semantic memory demands, whereas low alpha signifies a broad attentional demand not specifically tied to the task at hand (Klimesch, 1999). As well, the increased beta amplitude at occipital and frontal regions observed in individuals with trait-anxiety might contribute to decreased cognitive performance (Gordeev, 2007; Price and Budzynski, 2009).

**FIGURE 1:**
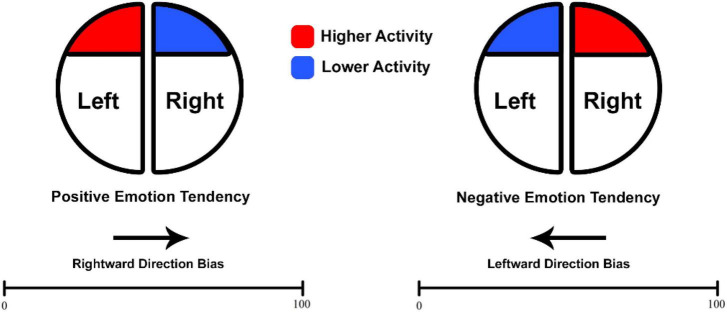
Expected association between baseline frontal asymmetry and direction bias. Adapted and modified from Vallortigara (2018) for the purpose of the current work.

In the present work, EEGs were recorded from participants at rest who were subsequently directed to complete a NP task. In keeping with the hypothesis of a prominent role of frontal EEG asymmetry in emotional processing, the main purpose (see Figure 1) of the present work was that higher baseline activation in the left hemisphere (identified through decreased alpha and/or increased beta) would correlate with a tendency toward rightward bias or, at minimum, a reduced leftward bias commonly observed in the spatial-numerical associations (Göbel et al., 2006; Loftus et al., 2008; Rotondaro et al., 2015). Moreover, building upon the role of EEG measures in cognitive processing, the investigation also aimed to evaluate their relation to absolute errors and reaction times (RTs) in MNL performance. Overall, the exploration of distinct oscillations intends to uncover their implications with respect to the accuracy and speed of MNL performance.

## 2 Materials and methods

### 2.1 Participants

A total of 76 university students in psychology participated in our study. To ensure consistency in handedness, we specifically recruited individuals who scored above 40 on the Edinburgh Handedness Questionnaire (EHQ) and were exclusively right-handed. All participants had a mean handedness score (scaled from −100 to 100) (Oldfield, 1971) of *M* = 80.4, *SD* = 13.7, possessed normal or corrected-to-normal vision, and had no history of neurological or psychiatric conditions. Data from five participants were omitted from the final analysis due to considerable EEG artifacts. Ultimately, data from 71 participants (41 Females; Mean age = 24.9; SD = 8.5) were utilized for the conclusive statistical analysis. The local Ethical Committee (Isfahan Ethical Council, date: 2023/05/29) approved the study, adhering to the ethical principles outlined in the Declaration of Helsinki concerning human subjects. Prior to participation, all individuals provided informed consent and received academic credit as compensation for their involvement.

### 2.2 EEG recording and analysis

**FIGURE 2:**
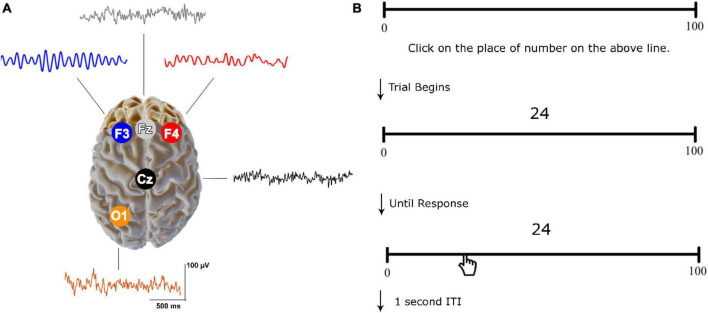
Resting-state EEG recordings and CNP task. **(A)** Electrode sites with sample raw signals. **(B)** An example of the trials used in CNP. The participants were asked to click on the place of the numbers displayed above a 25-cm line. Each number was randomly presented from a list.

As part of the ClinicalQ assessment, EEG recordings were obtained at locations F3, F4, Fz, Cz, and O1 (International 10–20 system for EEG site locations) (see Figure 2A) (Swingle, 2008; Swingle and Psych, 2016). The EEG Suite SA7900 version 6.7.1 by BioGraph Infinity (Thought Technology) and the ProComp 2 amplifier were employed to record, filter, and manage the EEG data. A signal sample rate of 256 Hz was utilized, while electrode impedance remained under 10 KΩ. The continuous EEG was processed with a fast Fourier Transform (FFT) and the band-pass filter of 0.1 to 60 Hz and a notch filter of 50 Hz were selected. Offline data analysis was carried out using EEGLab ver. 2019 (Delorme and Makeig, 2004). Instances of eye-related artifacts were managed through Independent Component Analysis (ICA), where independent components related to horizontal and vertical eye movements were visually identified and removed for each participant. Additionally, the EEG data for each participant underwent visual inspection to further eliminate any remaining artifacts. The EEG recordings were conducted for a minimum of 45 s for each electrode, ensuring artifact-free data collection, in accordance with reliability and validity standards for EEG/MEG frequency assessments (Thatcher, 2010; Wiesman et al., 2022). To obtain amplitude within specific bands, we used the FFT method focusing on absolute values (magnitude) and then averaged the amplitude values across relevant frequency bins for each band.

The brainwave frequencies for assessment included Theta (3–7 HZ), Alpha (8–12 Hz), Sensory Motor Rhythm (SMR) (13–15 Hz), Beta (16–25 Hz), HiBeta-Gamma (28–40 Hz), Lo-Alpha (8–9 Hz), and Hi-Alpha (11–12 Hz). All recordings were obtained at resting state in eye-closed condition. Participants were instructed to maintain relaxation without drifting into sleep, and avoid focusing on any specific thoughts. The EEG recording persisted for 5 min and consistently took place prior to engaging in the MNL task. The theta, alpha, and beta amplitude values were measured at F3 and F4 electrodes for the computation of an asymmetry score by comparing the activity in the right hemisphere (RH) to that in the left hemisphere (LH). The percentage of frontal asymmetry [Right Mean Amplitude (F4) – Left Mean Amplitude (F3) / MIN Amplitude (F3, F4) × 100] was computed to evaluate the relevant cortical activity. Considering the inverse correlation between alpha amplitude and cortical activity (Oakes et al., 2004), a positive alpha asymmetry index indicates greater activity in the left frontal region compared to the right, while a negative asymmetry index indicates higher activity in the right frontal region and lower activity in the left frontal cortical area. In contrast, a positive beta asymmetry index exhibits greater activity in the RH compared to LH, while a negative index reveals an oppositive pattern of activity. The TBR and LowAlpha/HighAlpha ratios were also calculated using quantitative EEG (qEEG) analysis (White et al., 2005). Determining the individual Alpha Peak Frequency (APF) involved analyzing EEG data to identify the specific frequency within the alpha band that exhibited the highest power for each individual participant at electrode Fz.

### 2.3 Computerized number-to-position (CNP) task

The research used a computerized number-to-position (CNP) task to measure the accuracy and bias of number line estimation (Siegler and Opfer, 2003; Nazari et al., 2021; Sabaghypour et al., 2023). The CNP task was programmed using the PsychoPy software v2022.1.2 (Peirce et al., 2011). Stimuli were presented in black on a white background. Participants completed the task comfortably in a dimly lit room, viewing a 17-inch monitor placed 60 cm away (Laptop Model G510 Lenovo; OS: Windows 10). The task involved 31 numerical stimuli presented two times on a 0–100 line (4, 6, 8, 12, 14, 17, 18, 21, 24, 25, 29, 33, 39, 42, 48, 50, 52, 54, 55, 57, 61, 64, 68, 72, 75, 79, 81, 84, 90, 93, 96) (Nazari et al., 2021). Each number was randomly displayed 2 cm above the center of a 25-cm line labeled “0” on the left and “100” on the right (Figure 2B). Participants clicked the mouse to respond, moving through the trials until the position of all numbers was estimated. Before the main task, a practice block with five trials was provided.

### 2.4 Statistical analysis

The evaluation of MNL behavioral measures included the absolute error, direction error, and reaction times (RTs). The *absolute error* and *direction error* were calculated by subtracting each participant’s predicted number from the target number (the correct response). The resulting mean absolute error was derived from the unsigned value, representing the magnitude of the error regardless of its direction. The mean direction error was determined by the signed value outcome: Negative values indicated that the participant’s response was lower than the target number, signifying a leftward bias on the MNL. Conversely, positive values indicated that the participant’s response exceeded the target number, indicating a rightward bias on the MNL. Correlation analyses were conducted to examine the relationships between frontal asymmetry and specific EEG frequencies. Statistical analysis was carried out using GraphPad Prism version 8.4.3 for Windows, GraphPad Software, Boston, Massachusetts USA, www.graphpad.com.

## 3 Results

### 3.1 Asymmetrical oscillatory pattern

**FIGURE 3:**
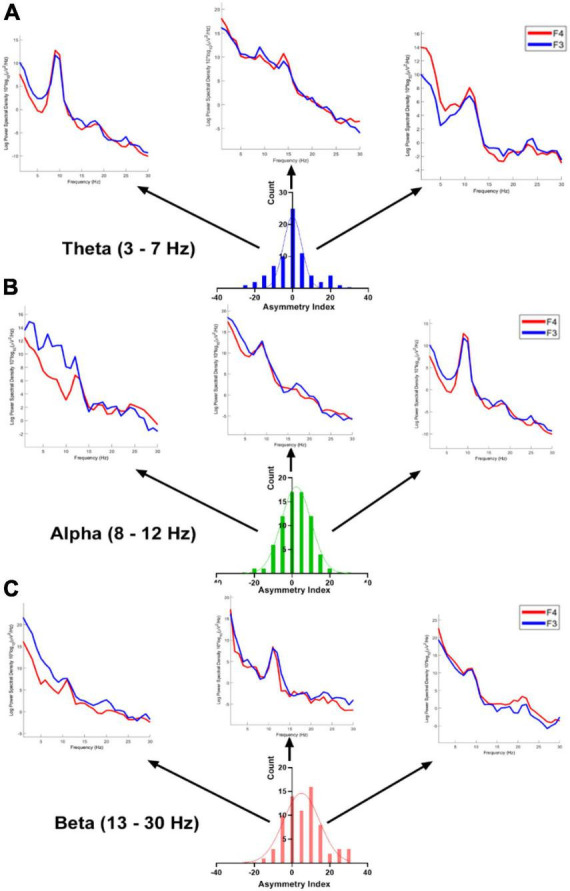
The distribution of asymmetry measures (% difference; bin = 5) across theta, alpha, and beta frequency bands at electrodes F3 and F4 was examined within our participants. Subplots **(A–C)** represent the distributions for theta, alpha, and beta frequency bands, respectively. The PSDs (Power Spectral Densities) depict individual sample data showcasing extreme (left and right) as well as middle values of the distributions.

Figure 3 illustrates the distribution of asymmetry measures observed within our participant population across distinct frequency bands (theta, alpha, and beta) at electrodes F3 and F4. Each subplot represents the distribution of asymmetry measures for a specific frequency band. The histograms depict the variation in asymmetry patterns observed for each frequency band separately.

### 3.2 Rs-EEG frontal asymmetry, direction, and absolute errors

**FIGURE 4:**
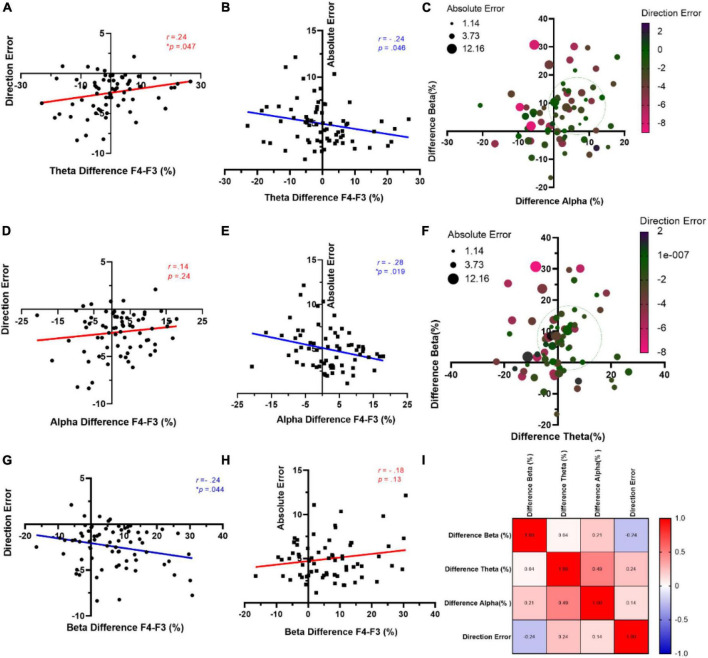
Correlation between behavioral measures and frontal asymmetry of rs-EEG. Relationship between mean amplitude of theta difference and direction error **(A)** and absolute error **(B)**. **(C)** Representation of both behavioral measures (direction and absolute errors) considering alpha and beta difference percent. **(D)** Direction error and alpha asymmetry. **(E)** Absolute error and alpha amplitude. **(F)** Representation of both behavioral measures (direction and absolute errors) considering theta and beta difference percent. **(G)** Beta difference and direction error. **(H)** Beta difference and absolute error. **(I)** Correlations matrix for the observed measures.

We utilized the Pearson correlation analysis, i.e., the potential predictability of mean directional and absolute errors, through an examination of frontal asymmetry across distinct frequency bands (specifically, theta, alpha, beta) at F3 and F4 electrodes. The results indicated significant positive and negative relationships between theta difference and direction error (*r* = 0.23, R^2^ = 0.056, *p* = 0.047) (Figure 4A) and absolute error (*r* = −0.24, R^2^ = 0.057, *p* = 0.046) (Figure 4B), respectively. Figure 4C shows a noteworthy reduction in both direction and absolute errors when there is a simultaneous occurrence range for positive alpha and beta differences. Moving to the alpha band, although there was no significant correlation between direction error and alpha difference (*r* = 0.14, R^2^ = 0.02, *p* = 0.254) (Figure 4D), a noteworthy negative correlation was observed between absolute error and alpha difference (*r* = −0.28, R^2^ = 0.08, *p* = 0.019) (Figure 4E). A similar pattern is evident in Figure 4F, where the coexistence of positive theta and beta differences in the approximate 0–20% range leads to a decrease in both types of errors. On the other hand, a negative trend emerged between beta difference and direction error (*r* = −0.24, R^2^ = 0.06, *p* = 0.044) (Figure 4G). However, there was no such significant relationship between beta difference and absolute error (*r* = −0.24, R^2^ = 0.06, *p* = 0.044) (Figure 4H). A correlation matrix between the above-mentioned measures is depicted in Figure 4I.

### 3.3 Cognitive measures of rs-EEG, absolute error, and RT

**FIGURE 5:**
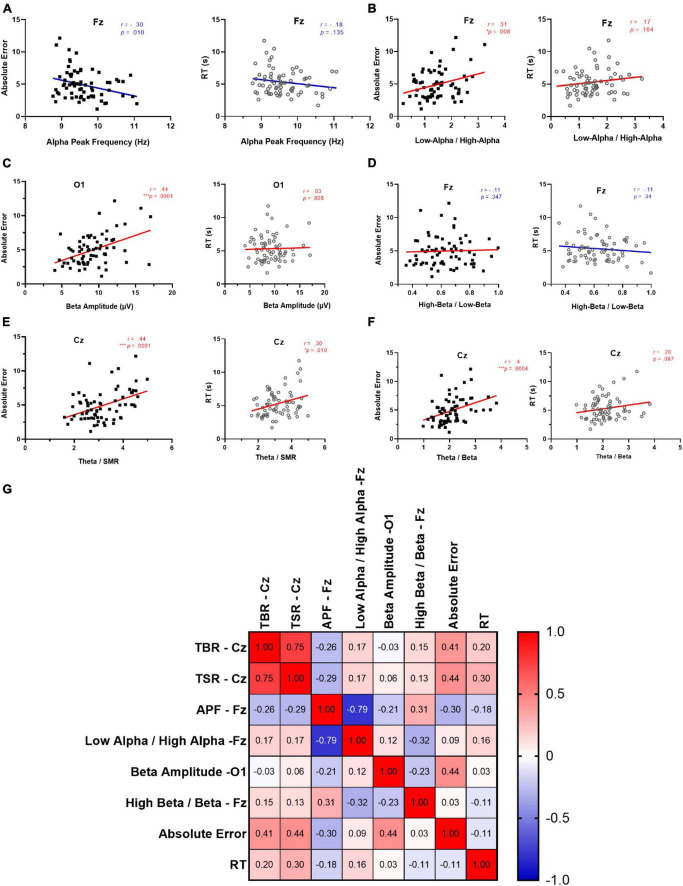
Correlation between behavioral indices and cognitive-related rs-EEG measures. **(A)** APF at Fz, absolute error (left panel), and RT (right panel). **(B)** lowAlpha/highAlpha at Fz, absolute error (left panel) and RT (right panel). **(C)** beta amplitude at O1, absolute error (left panel), and **(D)** RT (right panel). **(D)** High- beta / low- beta at Fz, absolute error (left panel). **(E)** TBR at Cz, absolute error (left panel) and RT (right panel), and **(F)** TSR at Cz, absolute error (left panel) and RT (right panel). **(G)** Colorations matrix between the above-mentioned measures.

An additional Pearson correlation analysis was used to investigate the potential predictability of behavioral indices (i.e., absolute error, and RT) with regard to other relevant rs-EEG measures. The results indicated significant negative relationships between APF at Fz and absolute error (*r* = −0.30, R^2^ = 0.090, *p* = 0.011) (Figure 5A, left). However, there was no such significant relationship between the alpha peak frequency and RT (*r* = −0.24, R^2^ = 0.06, *p* = 0.044) (Figure 5B, right). There were also significant positive relationships between low-alpha/high-alpha at Fz and absolute error (*r* = −0.31, R^2^ = 0.095, *p* = 0.008) (Figure 5A, left). However, there was no such significant relationship between the low-alpha/high-alpha and RT (*r* = −0.17, R^2^ = 0.03, *p* = 0.164) (Figure 5B, right). Moving to the beta band, the results indicated significant negative relationships between beta amplitude at O1 and absolute error (*r* = 0.44, R^2^ = 0.192, *p* < 0.001) (Figure 5C, left). However, there was no such significant relationship between the beta amplitude and RT (*r* = 0.029, R^2^ = 0.0008, *p* = 0.808) (Figure 5C, right). There were also no significant relationships between low- beta /high- beta at Fz and absolute error (*r* = 0.03, R^2^ = 0.001, *p* = 0.780) (Figure 5D, left). Similarly, there was no such significant relationship between the low- beta /high- beta and RT (*r* = −0.11, R^2^ = 0.01, *p* = 0.34) (Figure 5D, right).

Considering relative amplitude indices, there was a significant positive relationship between TSR at Cz and absolute error (*r* = 0.44, R^2^ = 0.192, *p* < 0.001) (Figure 5E, left) and between TSR and RT (*r* = 0.303, R^2^ = 0.092, *p* = 0.010) (Figure 5E, right). There were also significant positive relationships between TBR at Cz and absolute error (*r* = 0.41, R^2^ = 0.166, *p* < 0.001) (Figure 5F, left). Similarly, there was no such significant relationship between the TBR and RT (*r* = 0.20, R^2^ = 0.04, *p* = 0.087) (Figure 5F, right). A correlation matrix between the above-mentioned measures is depicted in Figure 5G.

## 4 Discussion

The present work investigated the rs-EEG regional characteristics and their correlation with mean direction error, mean absolute error, and RT in the CNP task. In our experiment, absolute errors indicated the extent of deviation of numerical magnitudes estimations, whereas direction bias indicated the deviation based solely on whether it is higher or lower than the expected value. Frontal asymmetry during rest is considered a trait variable with links to emotional processing (Paradiso et al., 1995; Jackson et al., 2000; Dalton et al., 2002; Zhang et al., 2020). Considering the recent insights highlighting the impact of emotions on MNL bias (Vallortigara, 2018; Sabaghypour et al., 2023), the idea of a potential correlation between asymmetry in frontal activity and direction bias was considered. Moreover, as a transfer of results from prior rs-EEG studies linking TBR, TSR, lowAlpha/highAlpha ratio, APF in centro-frontal regions to cognitive function (Klimesch, 1999; Sambrook and Goslin, 2015; Clarke et al., 2019; Finley et al., 2022), and the link between beta activity and trait-anxiety (Gordeev, 2007; Price and Budzynski, 2009), these rs-EEG measures were also examined.

First of all, since almost all our participants exhibited leftward direction errors, we replicated previous reports that neurologically healthy participants exhibit a general leftward bias in MNL tasks (Göbel et al., 2006; Longo and Lourenco, 2007; Loftus et al., 2009). Bowers and Heilman (1980b) observed that in healthy individuals, there was an inclination toward the left while performing line bisections (Bowers and Heilman, 1980b). They termed this tendency the “pseudoneglect” effect, which has also been referred to as “left-side underestimation” (Bradshaw et al., 1987) or “right hemispatial inattention” (Weintraub and Mesulam, 1988). A meta-analysis on pseudoneglect confirmed a moderate effect size for spatial pseudoneglect, establishing it as a reliable phenomenon (Jewell and McCourt, 2000). The prevailing hypothesis explaining this leftward bias involves differences in attention and activation between the right and left parietal hemispheres. Specifically, the leftward bias observed in healthy individuals is attributed to the allocation of attention toward the left hemispace, stemming from increased activation of the right hemisphere during spatial tasks (Jewell and McCourt, 2000). Prior research has proposed that performance in spatial-numerical association tasks might be influenced by various biases, including emotional valence (Sabaghypour et al., 2023). Regarding emotions, one perspective posits that the evidence supports a prefrontal neural index of valence (Jansari et al., 2000; Drake and Myers, 2006), implying a difference between positive and negative emotional traits.

With respect to hemispheric lateralization, the valence hypothesis of frontal asymmetry suggests that emotions are specialized and distributed between the left and right frontal hemispheres (Davidson, 1992). According to this hypothesis, the left frontal regions predominantly deal with positive motivations like hope and happiness (Harmon-Jones and Winkielman, 2007; Berkman and Lieberman, 2010; Cao et al., 2022). However, the right frontal regions are associated with vigilant attention that often occurs during certain negative affective states (Shackman et al., 2009; Grimshaw and Carmel, 2014). In contrast to the focus on parietal contributions in numerical representations (Hubbard et al., 2005; Knops et al., 2009), the significance of frontal cortical involvement in numerical cognition has been somewhat underappreciated. However, a bunch of recent research indicates that frontal contributions might hold particular importance in this research domain (Doricchi et al., 2005; Bachmann et al., 2010; Sabaghypour et al., 2023). Nevertheless, limited knowledge exists regarding the electrophysiological measures linking the individual variations with asymmetric orientation along the MNL.

Our findings on frontal asymmetry (F4-F3 difference) revealed significant positive correlations with MNL direction bias considering theta and significant negative associations concerning beta activity. Although there was an observed positive correlation tendency, the results did not reach statistical significance for alpha activity. Increased left frontal theta activity (negative difference percent in our measures) and increased right frontal beta activity (i.e., positive difference percent) have been reported to be linked to negative emotional traits (Coan and Allen, 2003; Dharmadhikari et al., 2018; Zhao et al., 2018; Koller-Schlaud et al., 2020). The correlation analysis of theta and beta in our results suggests that individuals exhibiting a larger positive theta (and larger negative beta) difference, which is correlated with positive emotional traits, displayed a greater inclination toward rightward attention, thereby moderating the general directional bias in MNL. These results are consistent with research that demonstrated shifts in emotions can affect attention with positive emotions inducing rightward bias, and negative emotions inducing leftward bias (Dimond et al., 1976; Levick et al., 1993). Our findings also align with the suggestion that the opposite side of the frontal cortex activity is linked to directing attention to the left and right side (Wright et al., 1995).

Furthermore, a significant negative and positive correlation between theta and beta asymmetries and absolute errors was observed, respectively. The reverse pattern regarding absolute errors might be attributed to a favorability toward positivity: Positive emotional traits have been linked to cognitive advantages, including increased cognitive flexibility, problem-solving abilities, and better decision-making (Isen, 2002). These results indicate that positive emotional traits could improve cognitive functions and promote effective cognitive performance (Fredrickson and Cohn, 2008).

Cognitive-related measures derived from rs-EEG data concerning mean absolute error and RT were explored to elucidate the possible correlation between brain activity patterns and performance in MNL. The findings revealed notable negative and positive correlations between APF and the low-alpha/high-alpha ratio with regard to absolute errors, respectively. APF detected through rs-EEG has been correlated to cognitive performance among healthy participants. APF that represents the frequency of the highest magnitude (or the center of gravity) within the range of 8–12 Hz and reflects a stable neurophysiological trait marker of cognitive preparedness (Angelakis et al., 2004; Grandy et al., 2013). A faster APF indicates increased magnitude (linked with improved cognitive performance) within the upper range of the alpha spectrum and vice versa. Our findings regarding APF and lowAlpha/highAlpha ratio, represent the first evidence linking this EEG index to numerical cognition, consistent with prior studies (Klimesch et al., 1990; Vogt et al., 1998; Klimesch, 1999; Rathee et al., 2020) that highlighted significant correlations between individual differences in APF and various cognitive measures. Theoretical explanations of these correlations suggest that alpha oscillations serve as a general mechanism for regulating neural inhibition (Klimesch et al., 2007) as well as controlling the flow of information within the brain (Mazaheri and Jensen, 2010), both of which contribute to improved cognitive performance.

Shifting focus to the beta frequency range, the results revealed a noteworthy negative correlation between beta activity at the occipital region (O1) and mean absolute errors in MNL. This negative relation might be interpreted in light of the fact that increased beta (especially in occipital areas with closed eyes) is linked with stress and anxiety traits (Gordeev, 2007; Díaz et al., 2019). Such results are also consistent with findings that linked anxiety traits to cognitive performance (Eysenck et al., 2007) including visuospatial (Eysenck et al., 2005) and numerical processing (Lindskog et al., 2017; Grežo and Sarmány-Schuller, 2018).

In terms of relative amplitude indices, significant positive correlations were observed between TSR and TBR at Cz with regard to the mean absolute error of participants. The observed significant positive correlations between TSR and TBR and absolute errors in participants’ performance align with findings suggesting a potential relationship between these ratios and cognitive performance in healthy adults (Putman et al., 2014; Clarke et al., 2019; Marlats et al., 2020). Few published studies explored the behavioral correlates of the TBR and TSR. In line with our findings, it has been evidenced that a high TBR/TSR is linked to a higher frequency of behavioral errors (van Dongen-Boomsma et al., 2010; Guleken et al., 2020). Furthermore, the TSR was the only significant measure that predicted the mean RTs. This finding aligns with previous research that reported an increase in central-midline TSR ratio could be linked to increased RT in behavioral tasks by recurrent lapses in attention (Ogrim et al., 2012).

To the best of our knowledge, this is the only study investigating the differential roles of rs-EEG measures on MNL performance. Set against the importance of our findings, it is essential to acknowledge the limitations of our research. Our study primarily focused on trait-related measures of frontal asymmetry, assuming relatively stable individual differences over time (Wheeler et al., 1993; Davidson, 1998). We speculated based on these trait asymmetries but did not include specific behavioral measures directly related to emotions. Future research might benefit from incorporating state-oriented inquiries to investigate changes in frontal EEG asymmetry in response to specific emotional states. Including behavioral measures such as the Positive and Negative Affect Schedule (PANAS) (Crawford and Henry, 2004) could offer deeper insights and more conclusive results into the observed relationships. Furthermore, it is essential to note that our study was constrained by limited resources, which impacted the extent of EEG data collection. The limited electrode coverage might have restricted our ability to capture more localized neural activity. Future research should consider employing a broader array of electrodes across various brain regions to obtain a more comprehensive understanding of relevant findings.

In light of these findings, the implications for future neuromodulation research are noteworthy. Since, successful performance in MNL tasks has been linked to mathematical achievements (Schneider et al., 2009; Simms et al., 2016), understanding the correlations between various EEG measures and performance in this task sheds light on potential avenues for utilizing neuromodulation approaches. Considering these findings, we shall also propose that employing techniques like transcranial alternating current stimulation (tACS) or TMS with a focus on theta or beta stimulation protocol might offer evidence supporting the causal role of frontal asymmetry in the direction bias.

## Data availability statement

The datasets presented in this study can be found in online repositories. The names of the repository/repositories and accession number(s) can be found below: doi: 10.17605/OSF.IO/FM8CJ.

## Ethics statement

The studies involving humans were approved by the local Ethical Committee approved the study, adhering to the ethical principles outlined in the Declaration of Helsinki concerning human subjects. Prior to participation, all individuals provided informed consent and received academic credit as compensation for their involvement. The studies were conducted in accordance with the local legislation and institutional requirements. The participants provided their written informed consent to participate in this study.

## Author contributions

SS: Conceptualization, Data curation, Formal analysis, Investigation, Methodology, Project administration, Resources, Software, Supervision, Validation, Visualization, Writing – original draft, Writing – review and editing. FN: Conceptualization, Data curation, Formal analysis, Investigation, Methodology, Project administration, Resources, Software, Supervision, Validation, Visualization, Writing – original draft, Writing – review and editing. NB: Formal analysis, Investigation, Project administration, Visualization, Writing – review and editing. FS: Data curation, Investigation, Project administration, Writing – review and editing. NZ: Data curation, Formal analysis, Investigation, Writing – review and editing.
